# The hidden limit in light: intrinsic noise reshaping Brillouin metrology

**DOI:** 10.1038/s41377-026-02248-y

**Published:** 2026-03-03

**Authors:** Leonardo Rossi, Gabriele Bolognini

**Affiliations:** Consiglio Nazionale delle Ricerche, Bologna, 40129 Italy

**Keywords:** Nonlinear optics, Fibre optics and optical communications

## Abstract

Spontaneous Brillouin scattering is widely used to probe the mechanical and thermal state of matter, yet it has been assumed to be intrinsically stable. Jin and colleagues overturn this view by showing that spontaneous Brillouin light carries its own thermally driven noise floor. Their framework predicts—and experiments confirm—a universal upper bound of SNR = 1 under ideal detection conditions which can become even more restrictive than the conventional shot-noise limit in practical Brillouin systems. This discovery introduces a new fundamental limit to Brillouin-based sensing, microscopy and metrology.

Brillouin scattering—both spontaneous (SpBS) and stimulated (SBS)—arises from the interaction of light with thermally induced density fluctuations in a medium. These fluctuations generate acoustic phonons which couple to the optical field and scatter incident photons into frequency-shifted components, known as Stokes and anti-Stokes waves, depending on whether their frequency is lower or higher than the pump light^1^. The Brillouin frequency shift is proportional to the speed of sound in the material, which in turn depends on physical properties such as compressibility, density, temperature, pressure, composition and heat capacity^1,2^. As a consequence, analyzing the Brillouin spectrum provides access to information about the mechanical and thermodynamic state of the medium.

Since its first theoretical and experimental description over a century ago^3^, Brillouin scattering has been exploited in a broad range of photonic technologies. In distributed optical fiber sensing, SpBS and SBS form the basis of temperature- and strain-sensing methods such as BOTDA and BOTDR^4–9^. In spectroscopy and microscopy, the sensitivity of Brillouin scattering to acoustic and viscoelastic properties enables contrast mechanisms not accessible through techniques primarily probing electronic or molecular transitions, giving rise to Brillouin spectroscopy and high-resolution Brillouin microscopy^2,10–12^. In integrated photonics, photon–phonon coupling has enabled microwave photonic processors, filters and Brillouin lasers^13,14^.

The performance of these systems hinges on precise spectral measurements, and therefore on maximizing the signal-to-noise ratio (SNR). Traditionally, noise in Brillouin metrology has been described in terms of thermal and shot noise, with shot noise seen as the fundamental limit. In SBS-based sensing, SpBS acts as an additional noise seed; for example, in BOTDA it introduces stochasticity in the gain process^15,16^. In SpBS-based systems, particularly BOTDR, noise studies have mainly focused on polarization- and phase-induced fluctuations, while the intensity of SpBS has been treated as statistically stable^4–9,17^.

A different perspective emerged in 1990, when Boyd, Rza̧ȩwski and Narum introduced a novel noise mechanism for SBS^18^. Earlier models assumed that SBS was initiated in a thin region near the fiber end^19^. In contrast, Boyd and coauthors showed that SBS could be initiated by thermally driven fluctuations distributed along the entire medium, producing large stochastic variations in the Stokes output even under highly stable continuous-wave excitation. These predictions were experimentally confirmed shortly thereafter by Gaeta and Boyd^20^, but only in the strongly stimulated regime, where the single-pass Brillouin gain G is much larger than unity (G ≈ 23–70).

However, most practical Brillouin metrology systems operate in the spontaneous regime, where G ≪ 1 and no amplification occurs. The effect of thermally driven fluctuations on SpBS statistics in this regime remained unknown for over three decades. The key questions were: do intrinsic thermal fluctuations introduce measurable intensity noise in SpBS, and does this impose a fundamental SNR limit? In a recent article in Light: Science & Applications^21^, Simeng Jin, Shuai Yao and co-workers revisit noise initiation and extend it to SpBS. By including Langevin thermal forces in the acoustic equation of the three-wave Brillouin model, they show that distributed thermal fluctuations generate a stochastic acoustic wave, which leads to intrinsic fluctuations in the spontaneously scattered Brillouin intensity. A schematic representation of this model is shown in Fig. 1, with inset (i) focusing on the description of Langevin noise.

**Fig. 1:**
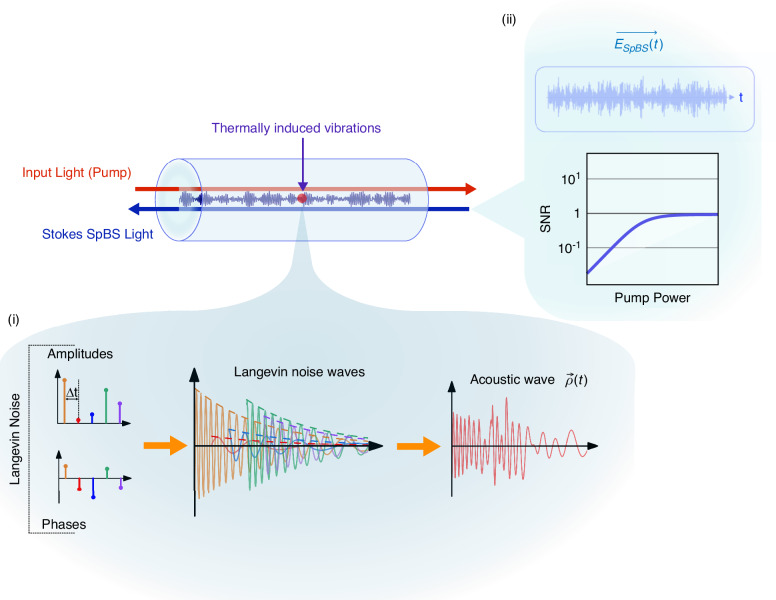
. **Scheme of the spontaneous** **Brillouin scattering model proposed in ref**. ^21^. $\vec{\rho }(t)$: complex amplitude of thermally induced acoustic vibration. ${\overrightarrow{{E}_{SpBS}(t)}}$: complex amplitude of SpBS field. The cylinder marks the light-matter interaction region, while the red and blue arrows represent the incident input light (or pump light) and the backward propagating SpBS Stokes light. (i): highlight of the presented model of the local excitation of acoustic waves by Langevin noise, defined as a series of damped acoustic waves with random amplitudes and phases, generated at discrete time intervals Δ*t*. The sum of the acoustic waves defines the localized acoustic field $\vec{\rho }(t)$, which in turn defines the random fluctuations of the Stokes light. (ii) shows the amplitude of the SpBS wave $\vec{{E}_{{SpBS}}}(t)$ and the evolution of the SNR as a function of the pump power, reaching an intrinsic limit of 1

A major result is the demonstration of a universal upper bound for SpBS SNR, shown in the inset (ii) of Fig. 1. Under ideal detection conditions—i.e., when the detection bandwidth is sufficiently large—the SNR of SpBS cannot exceed 1^21^. This mirrors the intuition from the stimulated-regime analysis^18^, but is now rigorously derived for the spontaneous regime and linked to practical system parameters. At higher input powers, this new limit even supersedes the shot-noise limit, which was up until now assumed to be the main limiting factor in Brillouin-based SNR.

The model also predicts how finite detection bandwidth, sampling interval and number of samples modify the apparent SNR. As the detection bandwidth increases beyond the intrinsic fluctuation bandwidth, the measured SNR decreases and converges to the intrinsic limit of 1. When it is narrower, only part of the fluctuation spectrum is detected, and the apparent SNR becomes >1. The authors experimentally validate these predictions using coherent detection in a 400 m polarization-maintaining fiber (G ≈ 0.23) and direct detection via a VIPA spectrometer (G ≈ 3.89). In both cases, the SNR increases with pump power but saturates at the predicted value. Control experiments replacing SpBS with CW light of equal power confirm that the saturation arises from intrinsic SpBS fluctuations.

The framework is then applied to three major Brillouin platforms. In Brillouin imaging, long interaction lengths and high powers make the system enter the SpBS-noise-limited regime, where increasing power no longer improves precision. In Brillouin microscopy, shorter interaction lengths and limited powers keep current systems shot-noise-limited^2,10–12^, but the framework predicts where future high-power implementations will encounter the SpBS limit. In BOTDR, the model shows that SpBS alone limits the single-pulse SNR to unity in polarization-maintaining fibers, while polarization fluctuations further reduce the ceiling in standard fibers^7,15–17^. This also explains why coding techniques that improve BOTDA performance^15,16^ offer limited benefits in BOTDR^17^.

Overall, the work of Jin, Yao and colleagues identifies intrinsic density fluctuations as a fundamental SNR limit in spontaneous Brillouin systems. Once a system operates in the SpBS-noise-dominated regime, increasing pump power cannot improve SNR; only longer averaging or design changes affecting fluctuation bandwidth can help. Similar thermally driven stochastic effects likely appear in other spontaneous scattering processes, such as Raman scattering.

By extending the concept of noise initiation from SBS to SpBS, and validating it experimentally, this work then provides new insight into the ultimate performance limits of Brillouin imaging, microscopy and distributed sensing.

## References

[1] Boyd, R. W. *Nonlinear Optics**.* 3rd edn (Orlando: Academic Press, 2008).

[2] Meng, Z. K. et al. Seeing cells in a new light: a renaissance of Brillouin spectroscopy. *Adv. Opt. Photonics* **8**, 300–327 (2016).

[3] Brillouin, L. Diffusion de la lumière et des rayons X par un corps transparent homogène - Influence de l’agitation thermique. *Annales de. Phys.* **9**, 88–122 (1922).

[4] Kurashima, T. et al. Brillouin optical-fiber time domain reflectometry. *IEICE Trans. Commun.* **E76-B**, 382–390 (1993).

[5] Shimizu, K. et al. Coherent self-heterodyne detection of spontaneously Brillouin-scattered light waves in a single-mode fiber. *Opt. Lett.* **18**, 185–187 (1993). 19802078 10.1364/ol.18.000185

[6] Mizuno, Y. et al. Proposal of Brillouin optical correlation-domain reflectometry (BOCDR). *Opt. Express* **16**, 12148–12153 (2008). 18679490 10.1364/oe.16.012148

[7] Motil, A., Bergman, A. & Tur, M. [INVITED] State of the art of Brillouin fiber-optic distributed sensing. *Opt. Laser Technol.* **78**, 81–103 (2016).

[8] Mizuno, Y. et al. Ultrahigh-speed distributed Brillouin reflectometry. *Light Sci. Appl.* **5**, e16184 (2016). 30167136 10.1038/lsa.2016.184 PMC6059889

[9] Yang, F., Gyger, F. & Thévenaz, L. Intense Brillouin amplification in gas using hollow-core waveguides. *Nat. Photonics* **14**, 700–708 (2020). 33824683 10.1038/s41566-020-0676-z PMC7610518

[10] Ballmann, C. W. et al. Stimulated brillouin scattering microscopic imaging. *Sci. Rep.* **5**, 18139 (2016). 10.1038/srep18139 PMC4686920 26691398

[11] Kabakova, I. et al. Brillouin microscopy. *Nat. Rev. Methods Prim.* **4**, 8 (2024). 10.1038/s43586-023-00286-z PMC11465583 39391288

[12] Scarcelli, G. & Yun, S. H. Confocal Brillouin microscopy for three-dimensional mechanical imaging. *Nat. Photonics* **2**, 39–43 (2008). 10.1038/nphoton.2007.250 PMC2757783 19812712

[13] Eggleton, B. J. et al. Brillouin integrated photonics. *Nat. Photonics* **13**, 664–677 (2019).

[14] Chauhan, N. et al. Visible light photonic integrated Brillouin laser. *Nat. Commun.* **12**, 4685 (2021). 34344891 10.1038/s41467-021-24926-8 PMC8333255

[15] Wang, S. et al. Study on the signal-to-noise ratio of Brillouin optical-time domain analyzers. *Opt. Express* **28**, 19864–19876 (2020). 32680057 10.1364/OE.393928

[16] Gao, X. et al. Impact of optical noises on unipolar-coded Brillouin optical time-domain analyzers. *Opt. Express* **29**, 22146–22158 (2021). 34265986 10.1364/OE.426655

[17] Jin, S. M. et al. Analytical signal-to-noise ratio model on frequency-scanned Brillouin optical time-domain reflectometry. *J. Lightwave Technol.* **42**, 5786–5796 (2024).

[18] Boyd, R. W., Rza̧ewski, K. & Narum, P. Noise initiation of stimulated Brillouin scattering. *Phys. Rev. A* **42**, 5514–5521 (1990). 9904689 10.1103/physreva.42.5514

[19] Zel’dovich, B. Y., Pilipetsky, N. F. & Shkunov, V. V. *Principles of Phase Conjugation* (Heidelberg: Springer, 1985).

[20] Gaeta, A. L. & Boyd, R. W. Stochastic dynamics of stimulated Brillouin scattering in an optical fiber. *Phys. Rev. A* **44**, 3205–3209 (1991). 9906321 10.1103/physreva.44.3205

[21] Jin, S. M. et al. A framework for spontaneous Brillouin noise: unveiling fundamental limits in Brillouin metrology. *Light Sci. Appl.* **15**, 44 (2026). 41484079 10.1038/s41377-025-02115-2 PMC12764778

